# Case Report: Recurrent Deposition in Renal Allografts: A Rare Case of Fibronectin Glomerulopathy Overlooked in Native Kidneys

**DOI:** 10.3389/fgene.2022.839703

**Published:** 2022-06-14

**Authors:** Xiaona Wei, Xiangdong Wang, Rui Zhang, Peifen Liang, Bo Liu, Lin Wang, Shuling Yue, Xiaojuan Li, Wenfang Chen, Qiongqiong Yang

**Affiliations:** ^1^ Department of Nephrology, Sun Yat-sen Memorial Hospital, Sun Yat-Sen University, Guangzhou, China; ^2^ Department of Pathology, the First Affiliated Hospital of Sun Yat-Sen University, Guangzhou, China; ^3^ Department of Renal Pathology, King Medical Diagnostics Center, Guangzhou, China; ^4^ Cellular and Molecular Diagnostics Center, Sun Yat-Sen Memorial Hospital, Sun Yat-Sen University, Guangzhou, China

**Keywords:** renal allograft, fibronectin glomerulopathy, fibronectin 1 mutation, recurrent post-transplantation, phenotypic heterogeneity of FN1 mutation

## Abstract

Fibronectin glomerulopathy (FNG) is a rare inherited kidney disease characterized by extensive deposition of fibronectin in the glomeruli, especially in the mesangial and subendothelial regions. The disease progresses slowly and eventually leads to kidney failure in 15–20 years. Here, we report an interesting case. The patient presented with proteinuria and was diagnosed with immune complex–mediated glomerulonephritis, and lupus nephritis was suspected. This patient progressed to end-stage renal disease after 18 years and received an allogeneic kidney transplant. However, proteinuria recurred 27 months after kidney transplantation. The renal biopsy found extensive deposition in glomeruli, and the patient was diagnosed with FNG using mass spectrometry analysis and confirmed by immunohistochemistry in both the native and transplanted kidneys. Gene sequencing revealed that a missense mutation in the fibronectin 1 (FN1) gene caused reduced binding to heparin, endothelial cells, and podocytes and impaired stress fiber formation. The patient had stable renal function but persistent nephrotic proteinuria after 6 months of follow-up. Given the persistence of abnormal circulating fibronectin levels, FNG can relapse following renal transplantation. The circulating fibronectin deposits on grafts, and renal function progressively deteriorates after recurrence. Therefore, whether renal transplantation is an acceptable treatment for FNG is still debatable.

## Introduction

Fibronectin glomerulopathy (FNG) is an autosomal-dominant renal disease of unknown etiology. FNG was first reported in 1995 and was characterized by a large amount of fibronectin (FN) deposition in glomeruli (Strøm et al., 1995). The clinical course of FNG patients is similar to that of chronic nephritis patients, most of whom present with hematuria and proteinuria (Zhang et al., 2020). Almost half of FNG patients have hypertension at the time of diagnosis; approximately 25% of FNG patients receive renal replacement therapy due to end-stage renal failure (Strøm et al., 1995; Abt and Cohen, 1996; Wu et al., 2017). The renal function of FNG patients who have reached end-stage renal failure can be stable for 4 months–10 years after renal transplantation (Gemperle et al., 1996; Otsuka et al., 2012). Immunohistochemically, serum-derived FN is found to deposit in glomeruli and redeposit in the transplanted kidney. The fibronectin 1 (FN1) gene was identified as the gene responsible for FNG (Castelletti et al., 2008; Ohtsubo et al., 2016). However, the mechanism of the disease is unknown, and there is no standard treatment. Here, we report a case of fibronectin glomerulopathy that was overlooked in the native kidney, recurred, and was eventually diagnosed in the renal allograft.

### Case Introduction

**FIGURE 1:**
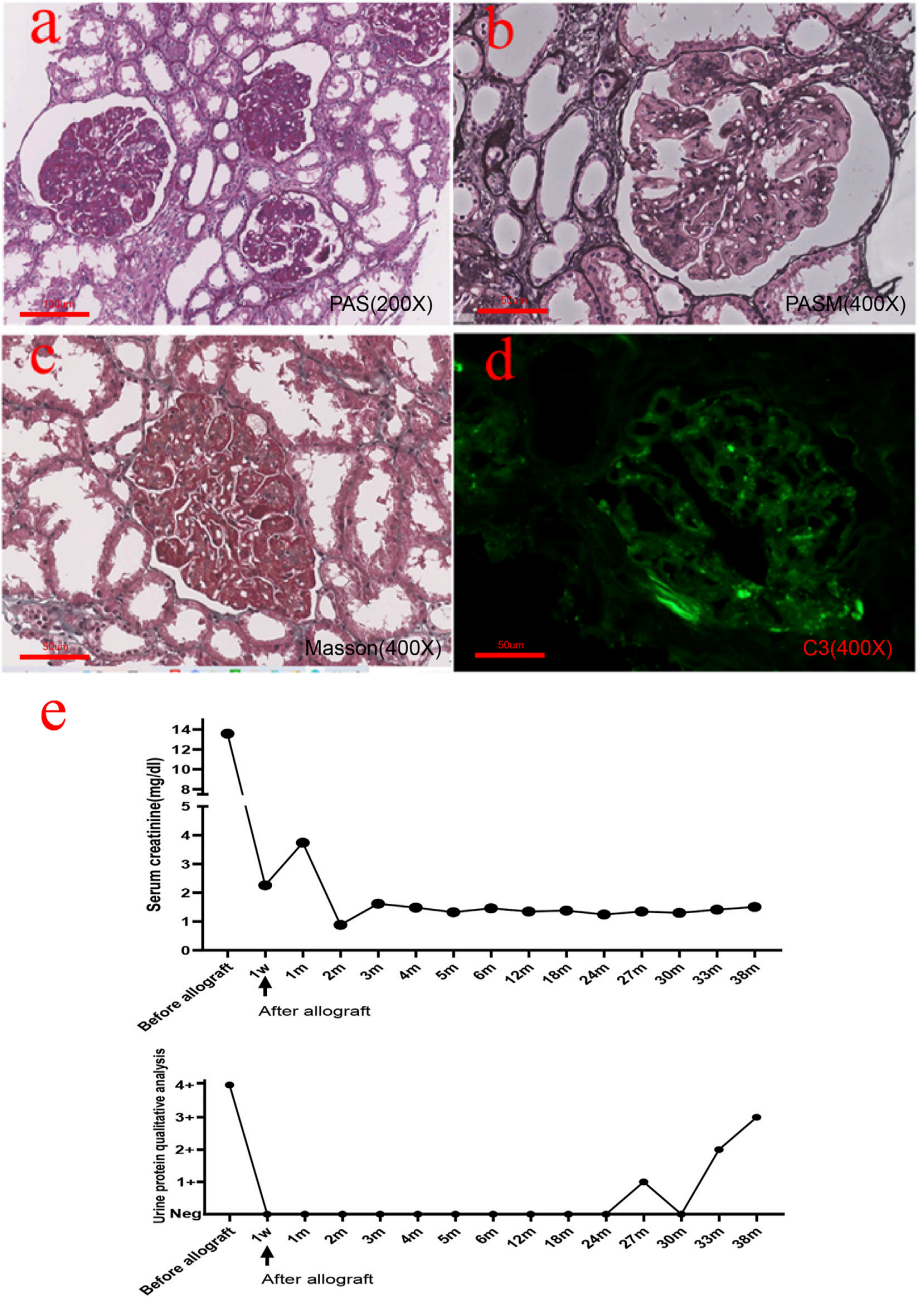
Histopathological findings from the native renal biopsy specimen. **(A,B)** Glomeruli showed moderate-to-severe mesangial hyperplasia and segmental interposition together with crescent formation in approximately 1/3 of the glomeruli. **(C)** Masson’s trichrome staining showed a large amount of deposition in the mesangial and subendothelial areas. **(D)** Immunofluorescence revealed mild C3 positivity. **(E)** Real-time changes in creatinine and urine protein post transplantation [**(A)**: 200×, **(B–D)**: 400×].

The patient was a 47-year-old female who complained of repeated episodes of lower extremity edema for 20 years. In 2001, the patient developed bilateral lower limb edema. The urinary test showed 4 + urine protein. Her renal function was normal, and her serum creatinine was approximately 0.92 mg/dl. A renal biopsy was performed. As shown in Figure 1, the glomeruli showed moderate-to-severe mesangial expansion and segmental interposition, and Masson’s trichrome staining showed a large amount of fuchsinophilic deposits in the mesangial and subendothelial areas. However, routine immunofluorescence found only trace immunoglobulin A (IgA), immunoglobulin M (IgM), and mild complement component 3 (C3) positivity. Immunoglobulin G (IgG) and complement component 1q (C1q) were both negative. Ultrastructural changes could not be evaluated because no glomeruli were found by electron microscopy. The patient was diagnosed with severe mesangial proliferative glomerulonephritis with membranous-like changes. Lupus nephritis was suspected due to a large amount of deposition viewed with Masson’s trichrome staining. However, during the subsequent 10-year follow-up period, antinuclear antibody (ANA), anti–double-stranded DNA antibody (anti-ds-DNA), and anti–Smith antibody (anti-Sm) remained negative, and no other systematic symptoms were found. The patient took glucocorticoids and cyclophosphamide orally, and the urinary test showed 3 + urine protein. The patient’s family history was negative for kidney disease.

In September 2014, she reached end-stage renal disease and received peritoneal dialysis and subsequently hemodialysis. In January 2018, the patient received an allogeneic kidney transplant from a donor after cardiac death (DCD). The preoperative creatinine level was 13.61 mg/dl. The operation was completed, and renal replacement therapy was terminated. The patient was treated with antirejection drugs (prednisone, mycophenolate mofetil, and cyclosporine). The patient’s serum creatinine reached the lowest value of 0.88 mg/dl 2 months post transplantation. One week after transplantation, the serum creatinine level and urinary protein showed a rapid decrease. Two months after transplantation, the level of serum creatinine had remained stable, and urinary protein was negative. Twenty-seven months after transplantation, the patient’s urine test showed protein 1+ and elevated serum creatinine 1.35 mg/dl (Figure 1E). Tripterygium wilfordii was recommended, but the response was poor, and renal function worsened. The laboratory test results of the patient during hospitalization are shown in Table 1.

**TABLE 1:** Laboratory results on admission.

Parameter	Value	Reference range
**Urine**		
pH	6.5	5.0–9.0
Red blood cells (/µl)	3	≤5
Urine specific gravity	1.010	1.005—1.030
Urine protein	2+	Negative
**Blood**		
Leukocyte count (10^9/L)	6.23	4.00–10.00
Hemoglobin (g/L)	110	115–150
Platelet count (10^9/L)	232	100–300
Urea nitrogen (mg/dl)	28.29	8.12–24.09
Creatinine (mg/dl)	1.48	0.60–1.30
Uric acid (mg/dl)	7.17	2.35–6.05
eGFR (ml/min per 1.73 m^2^)	41.81	≥90.0
Cystatin C (mg/L)	1.27	0.50–1.02
Total protein (g/L)	60.6	64.0–87.0
Albumin (g/L)	35.0	35.0–50.0
Sodium (mmol/L)	142	135–145
Potassium (mmol/L)	3.77	3.50–5.30
Chloride (mmol/L)	109	96–110
Calcium (mmol/L)	2.3	2.10–2.60
Phosphate (mmol/L)	1.39	0.97–1.62
Hemoglobin A1c, (%)	5.70	4.40–6.40
C3 (g/L)	0.82	0.79–1.17
C4 (g/L)	0.21	0.17–0.31
IgG (g/L)	7.43	10.13–15.13
IgA (g/L)	1.89	1.45–3.45
IgM (g/L)	3.69	0.92–2.04
Abbreviation: eGFR, estimated glomerular filtration rate; C3, complement component 3; C4, complement component 4		

**FIGURE 2:**
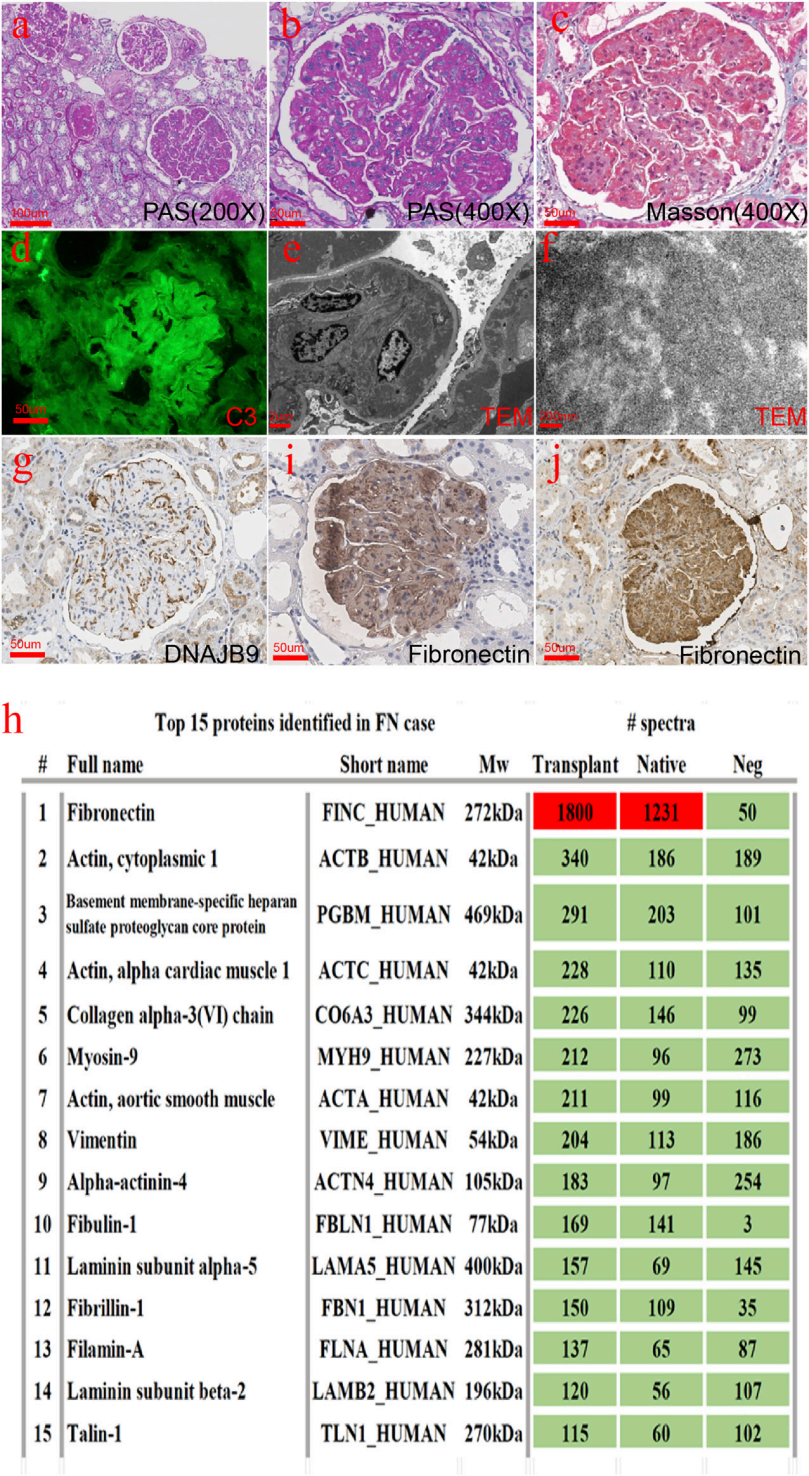
Histopathological findings from the transplanted renal biopsy specimen. **(A–C)** Glomeruli were enlarged and lobular. There was a large amount of deposition in the mesangial and subendothelial areas together with a slight proliferation of mesangial cells. **(D)** C3 was positive with an exudative pattern in immunofluorescence. **(E,F)** Deposition showed an obscure granular or short fibrillar appearance with a much higher electron density. **(G)** DNAJB9 immunohistochemistry turned negative. **(H)** Fibronectin was the most abundant protein in both the native and transplanted kidneys. **(I,J)** Fibronectin immunohistochemistry staining in the native kidney **(C)** and the transplanted kidney **(D)** was strongly positive [**(A)**: 200×, **(B–D)**, **(G)**, **(I–J)**: 400×].

At this point, we suspected that the patient’s initial diagnosis may have been wrong. To confirm the diagnosis, we performed a renal biopsy of the transplanted kidney under ultrasound. As Figure 2 shows, glomeruli were enlarged and lobular. There was a large amount of deposition in the mesangial and subendothelial areas together with a slight proliferation of mesangial cells. The subepithelial area was spared. The deposition was not as smooth and homogenous as seen with immune complex deposition but had slightly granular or coarse features. Eight out of 27 glomeruli developed global sclerosis, and two had segmental sclerosis. The pathological changes in the glomeruli were identical to those in the native kidney. Focal tubular atrophy and interstitial fibrosis were found in accordance with glomerular sclerosis. No signs of acute or chronic rejection, such as glomerulitis, tubulitis, interstitial infiltration, a double contour of the glomerular basement membrane, or multilayer renal tubular epithelial cells, were found. Arteriolar intimal fibrosis and hyalinosis were mild. There were six glomeruli in the immunofluorescence sample, among which two showed glomerulosclerosis and IgM, C3, and C1q were positive with an exudative pattern. IgG, IgA, and fibrinogen levels were negative. Complement component 4 d (C4d) was positive in less than 5% of the peritubular capillaries. IgG1, IgG2, IgG3, IgG4, and anti–phospholipase A2 receptor (PLA2R) antibodies were all negative. The deposition showed an obscure granular or short fibrillar appearance with a much higher density, which was different from what is usually observed with immune complex deposition. Fibrillary glomerulopathy was suspected, but DNAJB9 immunohistochemistry was negative (Figure 2G). The amount of deposition did not match the intensity of the immunofluorescence. Therefore, special proteins other than immunoglobulin and complement were suspected. Laser microdissection and mass spectrometry were performed to identify the nature of the deposition.

Briefly, 10 dissected glomeruli from 7-μm-thick FFPE sections from both the native and the transplanted kidney were collected using the LMD6 laser microdissection system (Leica). Two kidney transplant donor samples were used as controls. The dissected glomeruli were collected separately into 40 μl of Tris-EDTA buffer and 0.5% sodium deoxycholate detergent. After centrifugation at 10000 g for 1 min, glomerular fragments were incubated at 98°C for 60 min and sonicated with an ultrasonic cleaner at room temperature for 30 min. Solubilized proteins were further digested into peptides by sequencing-grade trypsin (Promega) overnight. The peptide was desalted with a C18 ZipTip (Millipore) and vacuum-dried. The sample was redissolved in 0.1% TFA and subjected to LC‐MS/MS with an Ultimate 3000 RSLC nanoLC coupled online with a Q Exactive mass spectrometer (Thermo Scientific). The resulting raw data were converted to mgf files with ProteoWizard and searched by the algorithm Mascot (Matrix Science). The PSM (peptide–spectrum match) hit was rescored by Percolator, and information on the proteins was summarized with a homemade script. The results were recombined and assigned probability scores of proteins and peptides in Scaffold software. According to the mass spectrometry results (Figure 2H), we stained the fibronectin of the native and transplanted kidneys. The immunohistochemical results showed that fibronectin was diffused and strongly positive (Figures 2I,J).

**FIGURE 3:**
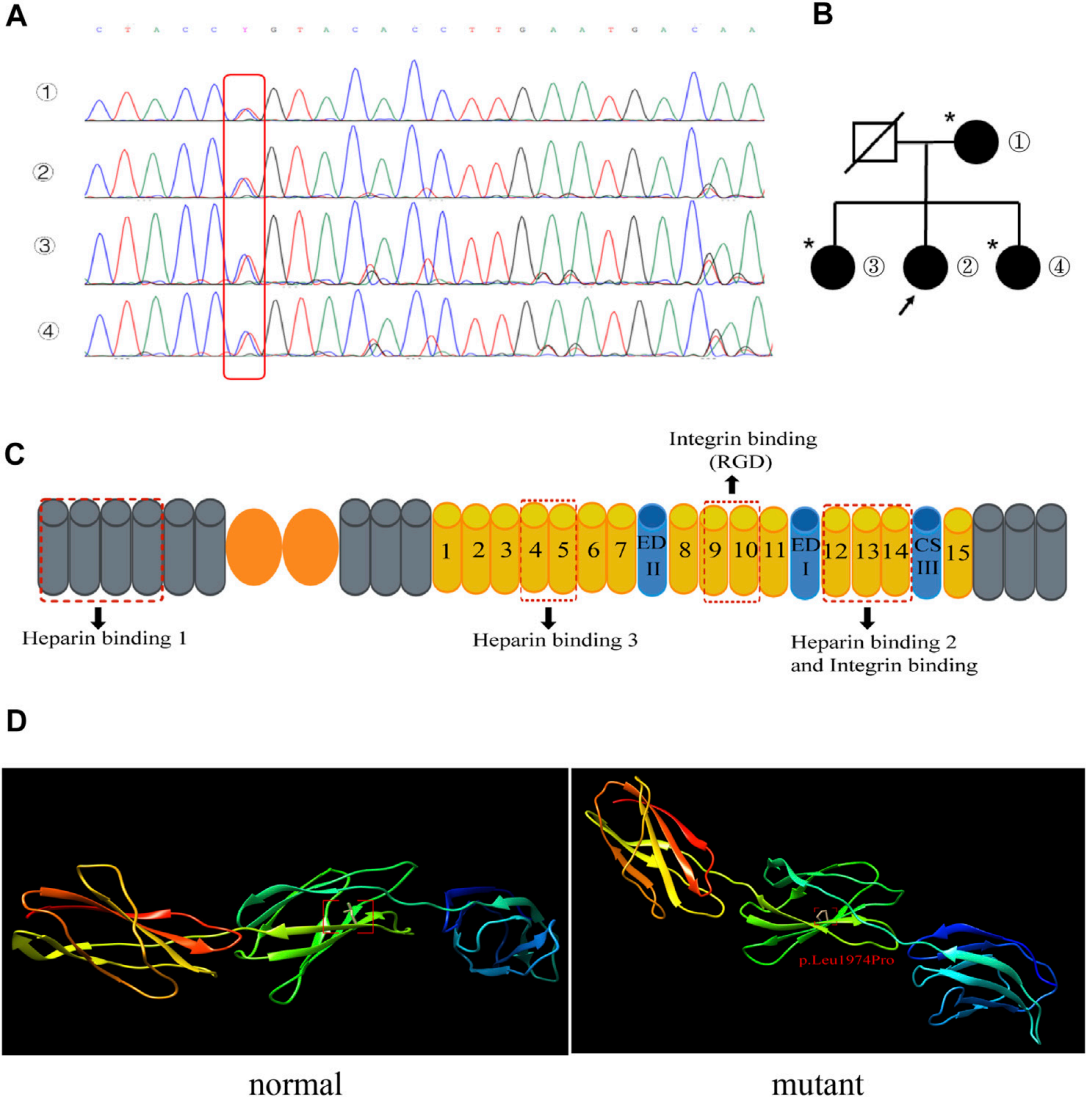
**(A)** Mutation T→C in the fibronectin gene in the family (red box): ① patient’s mother, ② the patient, and ③ and ④ the patient’s sisters. **(B)** Pedigrees of the family with the FN1 mutation. The arrow indicates the patient. An asterisk indicates a mutation site but no phenotype. **(C)** Schematic diagram of fibronectin. The fibronectin monomer consists of type I (gray), II (orange), and III (yellow) repeats and the alternatively spliced sites EDI, EDII, and IIICS. The three main heparin-binding domains and the binding sites for integrins are shown. **(D)** Normal (left) and mutant (right) fibronectin structures are shown. The p. Leu1974pro mutation introduced a neutral amino acid very close to the hydrophobic core of hep II.

In order to identify the mutation site of the patient’s FN1 gene, whole-exome gene sequencing was performed for the patient, and the results suggested that the subject was a heterozygous carrier of a missense variant of the FN1 gene on chromosome 2, NM_212,482.3:c.5921T> C:p.Leu1974Pro. Specifically, whole-exome gene sequencing showed that the nucleotide at position 5,921 of the FN1 gene changed from T to C, resulting in the translation of a protein with a proline rather than a leucine at position 1974. We performed Sanger sequencing site verification on the patient’s first-generation relatives and confirmed that they all carried this variant at this ectopic site (Figure 3A and Supplementary material 1). Surprisingly, the patient’s relatives carried the FN1 gene variant without symptoms (Figure 3B). FN1 interacted with cells and extracellular substances through the binding sites of integrin, heparin, and heparan sulfate proteoglycan. Each FN1 monomer consisted of homologous modules classified as type I, II, or III repeats (Figure 3C). The C-terminal type III 12–14 repeat (HEP II) contains the main binding site of heparin, and type III repeat 13 accounts for approximately 98% of hep II activity (Barkalow and Schwarzbauer, 1991; Sharma et al., 1999). We predicted the domain structure of the fibronectin protein structure after mutation by Chimera software. As illustrated in Figure 3D and Supplementary material 2, we can observe obvious changes in the structure. The p. Leu1974Pro mutation introduced a neutral amino acid very close to the hydrophobic core of hep II.

Through genetic analysis, FNG with the specific genetic variant was diagnosed. After 6 months of follow-up, the patient’s current urine protein was 3+, serum urea nitrogen was 27.59 mg/dl, and creatinine was 1.64 mg/dl. She is currently receiving conservative treatment, including diet therapy, cyclosporine (75 mg/bid), losartan potassium (100 mg/qd), and β-receptor blockers (12.5 mg/bid).

## Discussion

FNG is a rare nonimmune autosomal-dominant glomerulopathy. The clinical symptoms of FNG are atypical, including proteinuria, microscopic hematuria, hypertension, and slowly progressive renal function damage (Zhang et al., 2020). The diagnosis mainly depends on renal biopsy. The pathological examination will have the following manifestations (Lusco et al., 2017): periodic acid–Schiff-positive material throughout the mesangium and immunohistochemistry demonstrating strong fibronectin positivity, mesangial and subendothelial deposits that are mostly granular with focal fibrils in electron microscopy, fibronectin staining within mesangial areas and along the glomerular basement membrane, and limited increase in mesangial cellularity that is also present along the glomerular basement membrane. Our patient’s initial manifestations were bilateral lower limb edema and proteinuria, accompanied by elevated blood pressure. Renal biopsy pathology showed mild-to-moderate mesangial hyperplasia. Masson’s trichrome staining showed a large number of deposits in the mesangial area and under the endothelium. Immunofluorescence showed only mild IgA, IgM, and C3 positivity. Because the clinical and pathological manifestations of the patient were not typical and ultrastructural evidence was lacking, the diagnosis was not clear. After more than 10 years of hormone and immunosuppressive therapy, the patient still progressed to end-stage renal disease and underwent allogeneic kidney transplantation. In native kidneys, the interval between proteinuria and renal function impairment was approximately 10 years. However, after renal transplantation, the disease progressed rapidly. Proteinuria and renal function impairment occurred only 27 months after renal transplantation. The effect of hormone and immunosuppressive therapy was poor. Therefore, we performed a pathological biopsy of the transplanted kidney, and the light microscope pathological manifestation was similar to that of the native kidney. We further identified that the deposited protein was fibronectin by mass spectrometry in both the native and transplanted kidneys. Because FNG is a rare familial autosomal-dominant disease, we performed whole-exome sequencing on the patient and confirmed that the patient carried a fibronectin gene mutation. Therefore, the patient was diagnosed with FNG. Ohtsubo et al. (2016) showed that the binding of a heparin-binding site mutant (p.leu1974pro) to heparin decreased, but the binding to integrin was not affected. *In vitro*, the binding ability of the recombinant fragment protein (p.leu1974pro) to immortalized mouse podocytes was also similar to that of the wild-type protein. Whether the heparin-binding site mutant (p.leu1974pro) can reduce endothelial cell proliferation and cytoskeleton reorganization needs more research. For our patient, although her relatives carried this FN1 mutation, they were asymptomatic. The heterogeneity in the family meant that no genotype–phenotype correlation was observed, so additional studies are needed to determine any such correlation.

At present, there is no treatment for FNG. It has been reported that some patients are treated with hormones and immunosuppressants, but some patients fail to be treated or relapse after drug withdrawal. Although it has been reported that prednisone can continuously induce the remission of proteinuria in FNG patients (Goldman et al., 2021), the treatment is mainly symptomatic. The treatment methods include the control of blood pressure and use of ACEIs/ARBs to reduce glomerular perfusion pressure and proteinuria. It has been reported that ARB treatment can turn proteinuria negative and stabilize renal function in FNG patients for nearly 2 years (Fan et al., 2021). If progression to ESRD occurs, renal replacement therapy, including hemodialysis, peritoneal dialysis, and kidney transplantation, can be performed. To date, dozens of renal transplantations have been reported to be performed due to the diagnosis of FNG (Gemperle et al., 1996; Fujigaki et al., 1997; Castelletti et al., 2008; Otsuka et al., 2012; Ohtsubo et al., 2016), and the risk of recurrence is uncertain. The earliest recurrence was diagnosed 1 year post transplantation (Otsuka et al., 2012). We reviewed the reported cases of recurrence after FNG transplantation, and the prognoses of patients varied (Table2). Some patients’ renal function was stable for 10 years. Similarly, we reviewed that our patient’s renal function was generally stable following the recurrence of the disease. Therefore, we believe that renal transplantation is a feasible treatment for FNG patients. Since FNG is a rare disease, a follow-up as for IgA nephropathy may not be feasible, but tracking experience *via* a registry may be helpful.

**TABLE 2:** Prognosis of the four reported cases of recurrence after FNG transplantation.

	Sex	Age (y)	Follow-up
Strøm, Erik H, et al. (1995)	Male	31	Recurrence after renal transplantation (time unknown).
Gemperle O, et al. (1996)	Male	46	Proteinuria recurred 7 months after transplantation (0.7 g/24 h), and the recurrence was confirmed by renal transplant biopsy 23 months after operation (urinary protein 2.57 g/24 h, serum creatinine 142 μmol/ml).
Castelletti F, et al. (2008)	Female	48	Recurrence occurred 3 years after the operation.
Otsuka Y, et al. (2012)	Female	52	Proteinuria began to appear 4 months after the operation. One year later, FNG was diagnosed with a renal transplant biopsy. (serum creatinine 136 μmol/ml, blood urea nitrogen 11 mmol/ml, and urinary protein 0.4 g/24 h).

FNG is a rare renal disease that can recur after renal transplantation. Currently, there is no effective treatment for this disease. Although proteinuria may relapse after transplantation, renal function may remain stable for many years. Renal transplantation is an acceptable choice for FNG patients.

## Data Availability

The original contributions presented in the study are included in the article/Supplementary Material; further inquiries can be directed to the corresponding authors.
