# Supplementary figures and images for: Case Report: Recurrent Deposition in Renal Allografts: A Rare Case of Fibronectin Glomerulopathy Overlooked in Native Kidneys
Source: Front Genet. 2022 Jun 14;13:839703. doi: 10.3389/fgene.2022.839703 (PMC9237440; doi:10.3389/fgene.2022.839703)

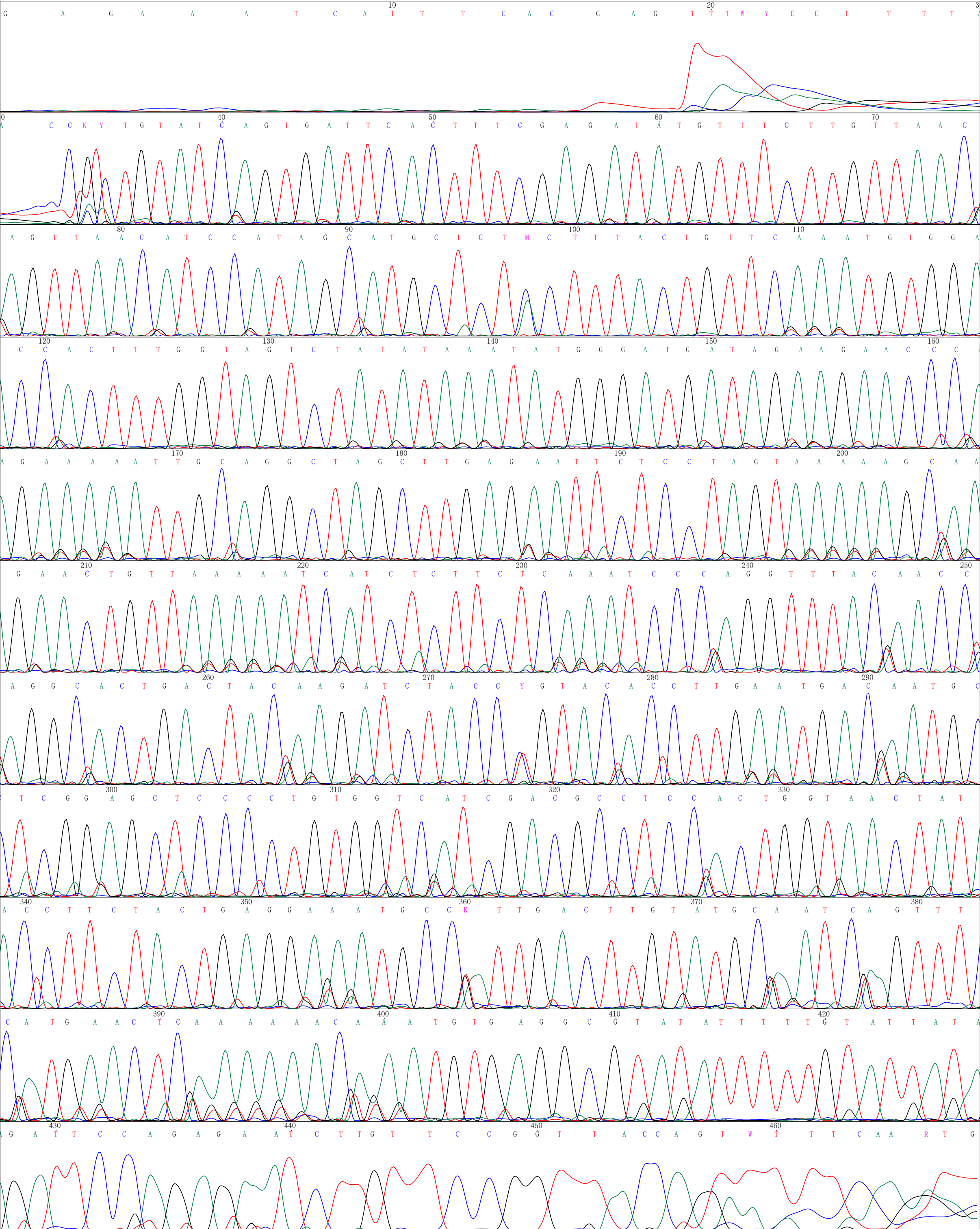

Supplement: Supplementary file 1 [file DataSheet2.PDF]

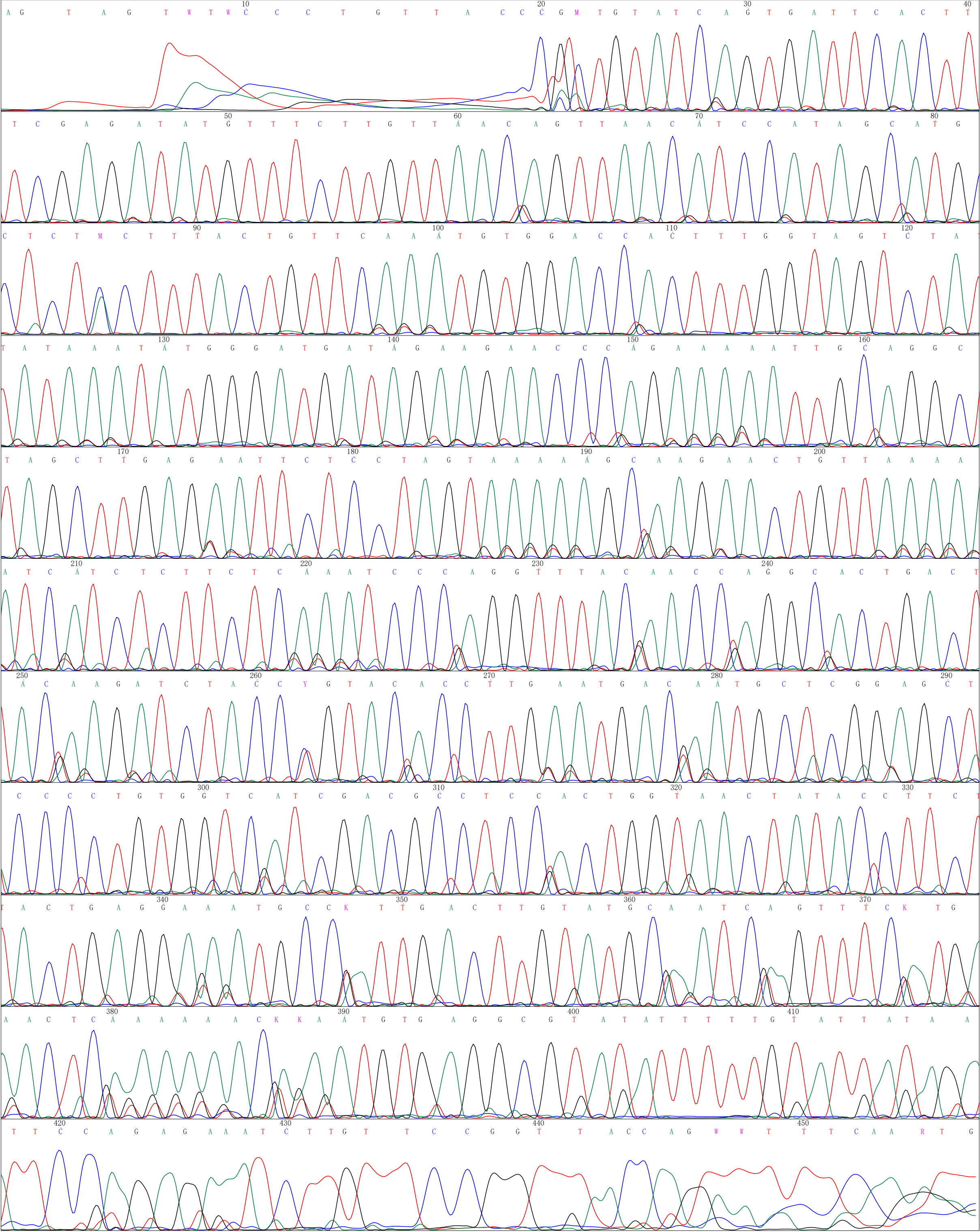

Supplement: Supplementary file 5 [file DataSheet1.PDF]
