# Supplementary material for: Case Report: Recurrent Deposition in Renal Allografts: A Rare Case of Fibronectin Glomerulopathy Overlooked in Native Kidneys
Source: Front Genet. 2022 Jun 14;13:839703. doi: 10.3389/fgene.2022.839703 (PMC9237440; doi:10.3389/fgene.2022.839703)
Supplement: Supplementary file 3 [file Table1.DOCX]

**Supplementary materials**

**Methodology**

**1. Histology and Immunostaining**

For histology, 2 μm paraffin-embedded kidney sections were subjected to hematoxylin-eosin (HE) staining, Schiff periodate (PAS) staining and Masson's trichrom staining (Masson) by the standard protocol. For immunostaining, 4 μm paraffin-embedded kidney sections were deparaffinization and rehydrated at room temperature. Sodium citrate solution at pH = 7.0 was used for antigen retrieval. Primary antibodies against Fibronectin (Abcam, Cambridge, UK) were incubated overnight at 4 ◦C and then incubated with secondary antibodies for 1 h at room temperature. Images were taken under an optical microscope (Olympus, Japan).

**2.** **Whole Exome Sequencing (WES)**

Approximately 2~3 ml of peripheral blood was extracted for DNA library construction. Illumina DNA Prep with exome Enrichment kit (Illumina, USA) was used according to the manufacturer’s protocol. Illumina Nextseq550 (San Diego, CA, USA) was used for 150bp paired-end sequencing. Each individual yield ~10GB of fastq data, with >97.5% of the targeted regions (exonic regions of all nuclear genes plus +/−20bp of exon-intron boundaries) sequenced more than 20 times, the average depth of coverage was ~150X.

The original fastq data was processed with our WES pipeline, and FastQC toolkit was used first to assess the quality of the sequencing, the reads were then aligned to the human reference genome hg19 using the Burrows-Wheeler Aligner (BWA). Picard tool was used to process BAM data and the realignment of the indel regions. We next used the Genome Analysis Toolkit (GATK) to recalibrate base qualities, variant detection and calling, and then outputted the variant call format (VCF) files. Then ANNOVAR was used to annotate the VCF files. We further filtered the variants by quality/coverage depth and minor allele frequency, and evaluated the variants based on their pathogenicity, patient’s phenotype, inheritance mode, and clinical significance.

For the purpose of assessing the variant carrier status of the proband's relatives, Sanger sequencing was used. The primers for FN1 variant c.5921T>C (NM_212482.3) was, forward primer (5'->3') TTTTCTCCCGAGCCGTTCTA, and the reverse primer (5'->3') ACCAGTGTCCTTGTCTGTATCA was used. Genomic DNA using polymerase chain reaction (PCR) and direct sequencing of FN1 was conducted.

**3. Normal and predicted mutant Fibronectin structures**

The crystal structures of the heparin and integrin-binding segments of human Fibronectin was downloaded from the Protein Data Bank (https://www.rcsb.org/structure/1FNH). The normal and mutated structures were by UCSF Chimera software. In Chimera, point mutation can select different amino acid structural states of point mutation according to the probability of different amino acid orientations (experimental data). And later through kinetic simulation, to adjust the changes of other amino acid structure after point mutation. The predicted mutant structure is finally obtained.
