# Supplementary material for: Case Report: Recurrent Deposition in Renal Allografts: A Rare Case of Fibronectin Glomerulopathy Overlooked in Native Kidneys
Source: Front Genet. 2022 Jun 14;13:839703. doi: 10.3389/fgene.2022.839703 (PMC9237440; doi:10.3389/fgene.2022.839703)

File: E12_17-00-30.ab1 Run Ended: Sep 28, 2021, 22:13:29 Signal C:12776 T:17043 A:18924 G:14015 Sequence: GM
Lane: 5 Base spacing 10.95 454 bases in 5531 scans Page 1 of 1

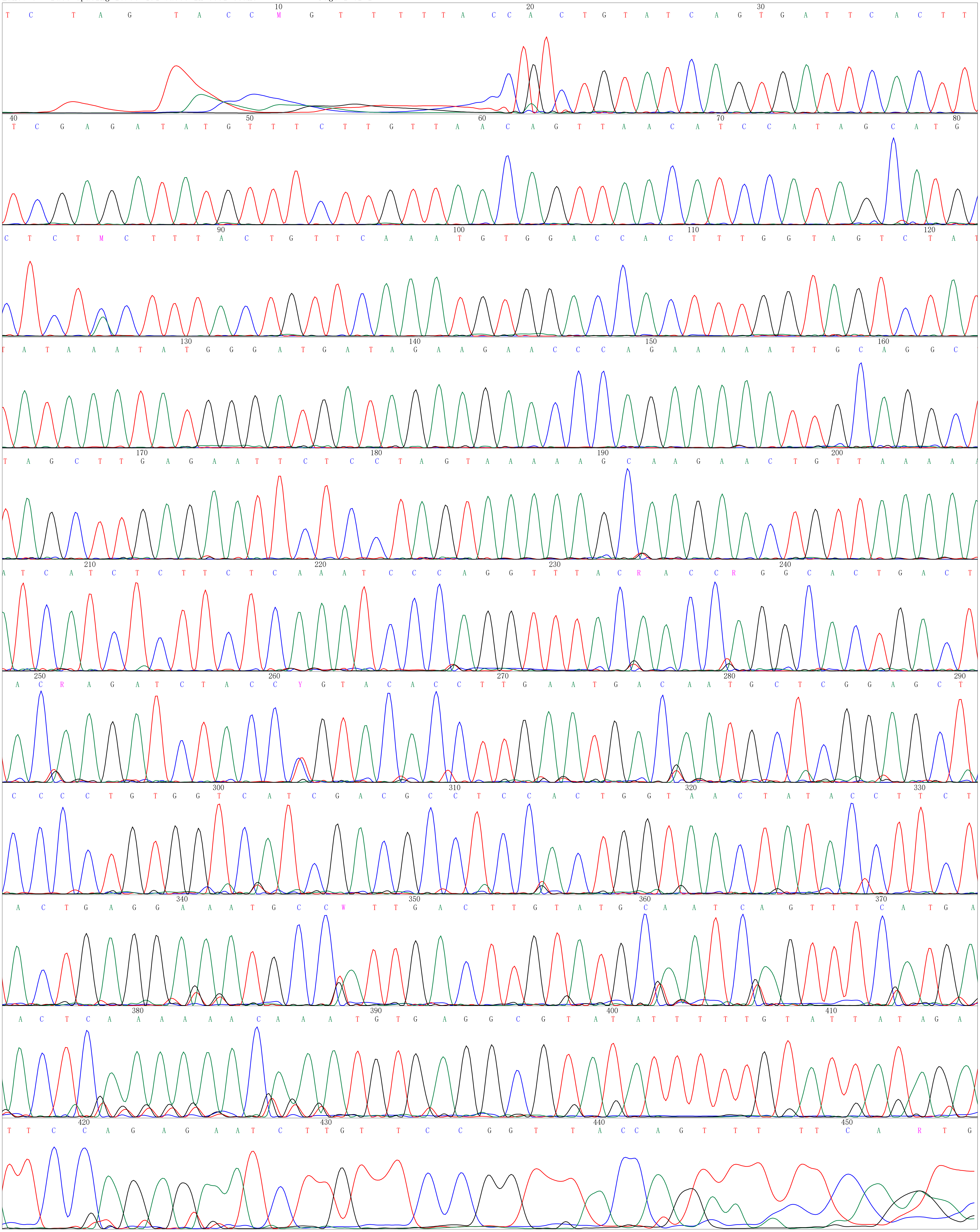

Supplement: Supplementary file 2 [file DataSheet4.PDF]
