# Supplementary material for: Case Report: Recurrent Deposition in Renal Allografts: A Rare Case of Fibronectin Glomerulopathy Overlooked in Native Kidneys
Source: Front Genet. 2022 Jun 14;13:839703. doi: 10.3389/fgene.2022.839703 (PMC9237440; doi:10.3389/fgene.2022.839703)

File: _F12_17-00-30.ab1 Run Ended: Sep 28, 2021, 22:13:29 Signal C:5505 T:6346 A:7316 G:4858 Sequence: SAG
Lane: 6 Base spacing 10.95 457 bases in 5559 scans Page 1 of 1

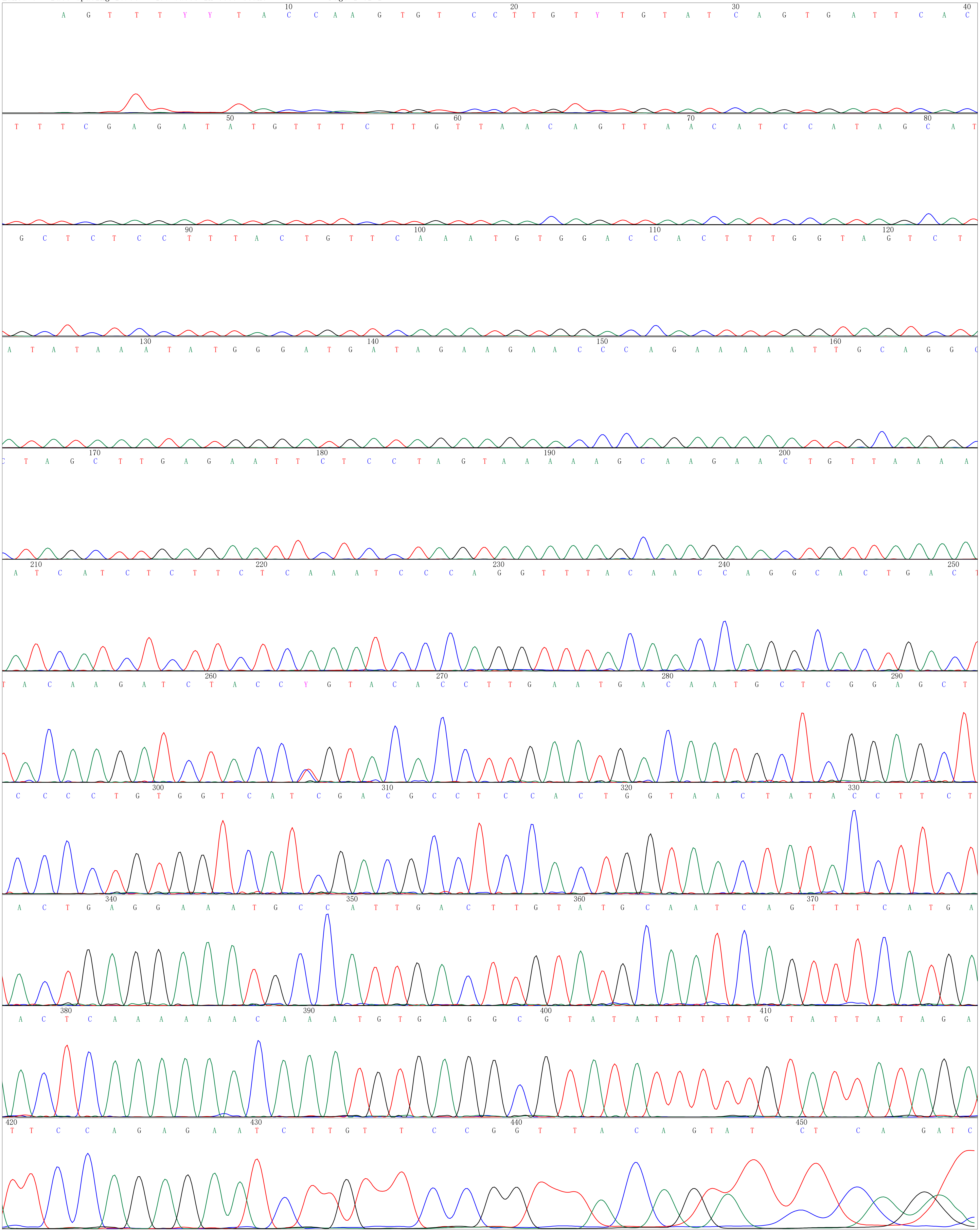

Supplement: Supplementary file 4 [file DataSheet3.PDF]
